# A Novel Multi-Port Containment System for Laparoscopic Power Morcellation to Prevent Tumoral Spread: A Retrospective Cohort Study

**DOI:** 10.3389/fsurg.2022.803950

**Published:** 2022-02-03

**Authors:** Wenhui Wang, Haiyan Liang, Fang Zhao, Huan Yu, Chunhong Rong, Weiwei Feng, Qingyun Chen, Yanjun Yang, Qian Li, Dingqing Feng, Yuxiao Dong, Ming Xue, Jing Liang, Bin Ling

**Affiliations:** ^1^Graduate School of Peking Union Medical College, Chinese Academy of Medical Sciences, Beijing, China; ^2^Department of Obstetrics and Gynecology, China-Japan Friendship Hospital, Beijing, China; ^3^Graduate School of Beijing University of Chinese Medicine, Beijing, China

**Keywords:** uterine leiomyoma, laparoscopic myomectomy, power morcellation, containment system, uterine sarcoma

## Abstract

**Objective:**

To report a novel multi-port containment (NMC) system for laparoscopic power morcellation to prevent tumoral spread and to evaluate its safety, validity, and feasibility.

**Methods:**

This retrospective study included women who underwent laparoscopic myomectomy (LM) between January 2014 and August 2020 at a single academic institution. The NMC system was used in the study group (n = 193); the control group underwent unprotected LM (*n* = 1753).

**Results:**

After 1:1 propensity score matching, no significant differences in the baseline characteristics were observed between 193 matched pairs. Bag damages were detected in two cases in the study group before morcellation, and the NMC systems were replaced. There were no significant differences between the two groups in terms of the complications, total operative time, estimated blood loss, or postoperative hospitalization duration. In the study group, all operations were completed and no system rupture or leakage was observed. The median follow-up times were 21 and 54 months in the study and control groups, respectively. There was no peritoneal tissue spread in the study group. However, three (3/5, 0.6%) and six (6/1,753, 0.3%) patients in the control group experienced malignant and benign peritoneal tissue spread, respectively.

**Conclusion:**

The NMC system for laparoscopic power morcellation is valid, safe, and feasible for preventing a tumor spread.

## Introduction

Uterine leiomyoma (UL) is the most common tumor of the female reproductive system, with an incidence of >70% (1). Laparoscopic myomectomy (LM) is the most frequent fertility-sparing or uterine-conserving procedure (2, 3). Power morcellation facilitates the efficient fragmentation and removal of tissues *via* small incisions (4). However, unprotective morcellation seriously violates the principle of the no-touch isolation technique and challenges operational safety, thereby becoming a worldwide concern (5, 6).

Laparoscopic power morcellation in the management of uterine malignancy, especially occult uterine sarcomas, may inadvertently cause disease upstaging and negatively affect the prognosis (6–8). In 2014, the U.S. Food and Drug Administration (FDA) reported that the incidence of occult uterine sarcoma was 1 per 352 individuals, and issued a black box warning about power morcellation (9). This was reiterated in 2020 and power morcellation for myomectomy was recommended only if performed with the containment system (10).

However, owing to the lack of compatibility between the containment system and laparoscopic instruments, the leakage rate of the bags is very high (11–13). A prospective multi-center study was paused due to blue dye spillage, mostly from the lateral puncture site, in 9.2% of the cases (12). In addition, bags render morcellation more cumbersome, resulting in increased operative times (14). The aim of this study was to report a novel multi-port containment (NMC) system for laparoscopic power morcellation for preventing tumor spread and to evaluate its safety, validity, and feasibility.

## Materials and Methods

**Figure 1:**
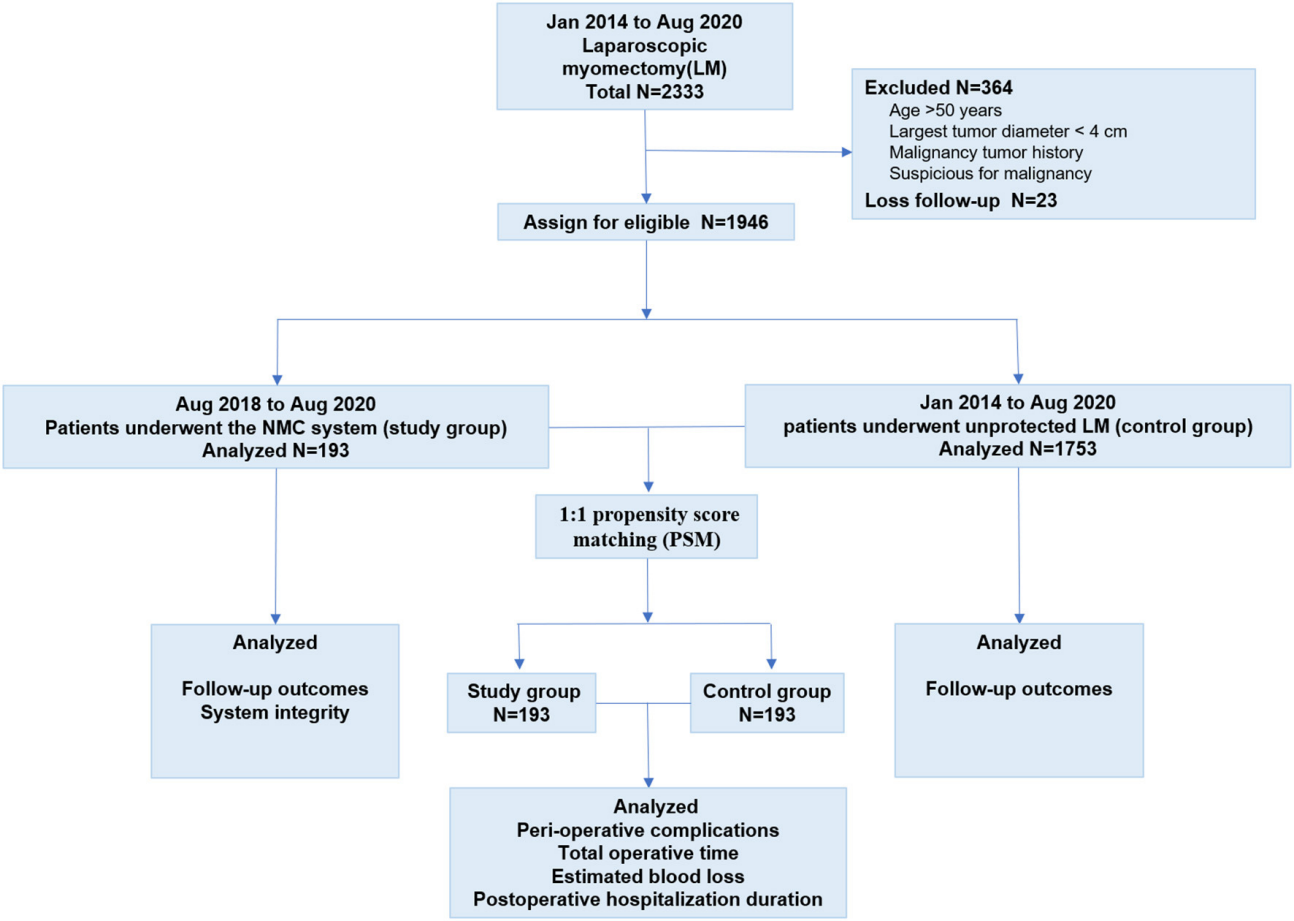
Study design.

The flow chart of the study design is presented in Figure 1. LM was performed with an NMC system in a total of 193 patients (the study group) from August 1, 2018 to August 1, 2020 at the Gynecology Center of the China-Japan Friendship Hospital in Beijing, China. Considering the stability of the LM technique and the large sample size required for comparison, 1,753 patients treated with unprotected LM from January 1, 2014 to August 1, 2020 were included in the control group. A total of 364 patients were excluded for the following reasons: (1) age > 50 years; (2) largest tumor diameter <4 cm; (3) history of malignancy; and (4) suspected malignancy. Another 23 patients were lost to follow-up, and were thus, excluded from the control group. All surgeries were performed by the same group of experienced surgeons (B.L., J.L., and H.L.); they were highly trained in minimally invasive procedures.

Demographic, perioperative, and follow-up data were collected and analyzed. The demographic data comprised age, body mass index (BMI), gravidity, parity, hypertension and diabetes status, the American Society of Anesthesiologists (ASA) score, hemoglobin level, major indications of LM (such as menorrhagia), intestinal and urinary tract disorders, presence of abdominal distention or pain, and fertility requirements. Data were also collected on the history of previous abdominal/pelvic surgeries; the sizes, numbers, and locations of the leiomyomas; and the pathological types of the lesions. Perioperative data comprised the integrity of the system after morcellation, intraoperative complications (including visceral, vascular, or nerve injuries; estimated blood loss > 1,000 mL; or serious anesthesia complications), postoperative complications (12), total operative time (defined as the time from incision to closure), estimated blood loss (defined as the surgeon's estimate recorded in the operative record), and postoperative hospitalization duration. All patients were followed up with ultrasound or magnetic resonance imaging at 1 month, 6 months, and 1 year after the operation.

All surgeries were performed by senior surgeons who followed the same standardized procedures for each intervention; the patients were placed under general anesthesia in a semi-lithotomy position. A standard four-port operative laparoscope was introduced for direct visual entry. During each surgical intervention, a careful and systematic inspection of the uterus, ovaries, and entire pelvis was performed. In the control group, standard intra-abdominal uncontained power morcellation was performed using a reusable, uterine power morcellator (KANGJI medical) (15). The NMC system was used for the study group.

**Figure 2:**
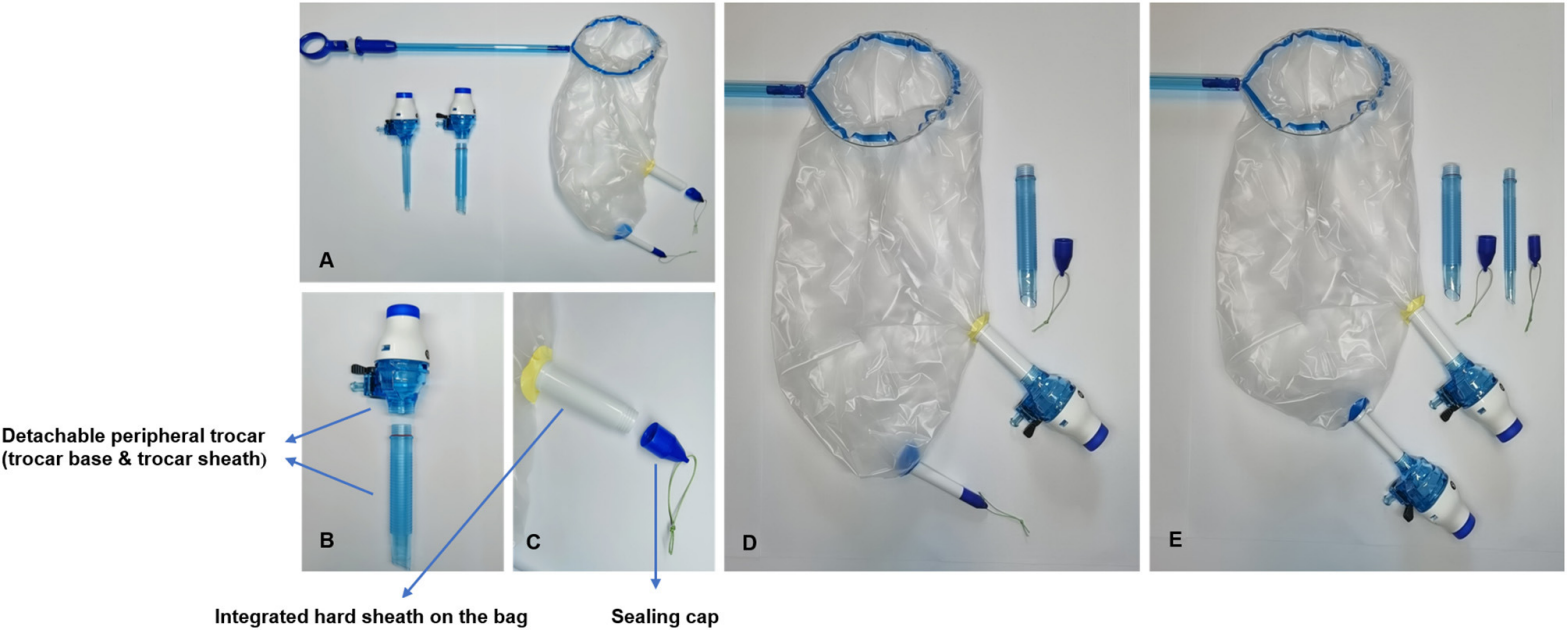
Main components of the novel multi-port containment (NMC) system. **(A)** The main components of the NMC system include the detachable peripheral trocar and the retrieval bag. **(B)** Detachable peripheral trocar (trocar base and trocar sheath). **(C)** Hard sheath on the bag and sealing cap. **(D,E)** Transposing sealing caps and trocar bases by threaded structures.

The main components of the NMC system are shown in Figure 2 and include a detachable trocar (trocar base and trocar sheath) and a retrieval bag (soft bag body, hard sheath on the bag, and sealing cap).

**Figure 3:**
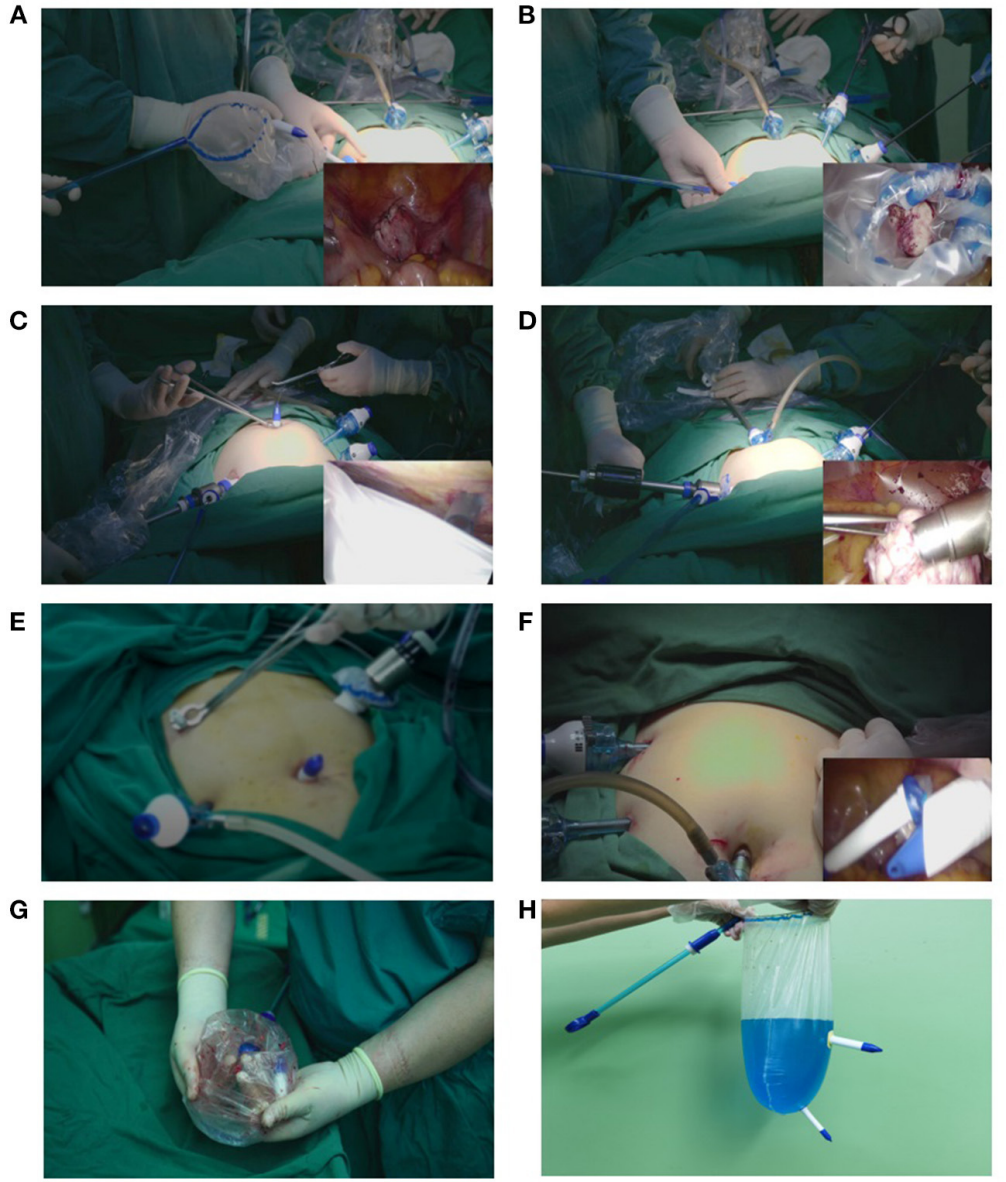
Placement and extraction of the novel multi-port containment (NMC) system. **(A)** The bag is placed into the abdominal cavity through the right lower 20-mm introduction sheath. **(B)** The myoma is resected into the bag via its large opening. **(C)** The sealing caps on the hard sheaths are unscrewed and replaced with trocar bases. **(D)** The abdominal cavity is de-sufflated, and simultaneously, a pseudo-pneumoperitoneum is established by inflating the bag via the umbilical trocar. Thereafter, power morcellation is performed. **(E)** After morcellation, the trocar bases are unscrewed and replaced with the corresponding sealing caps. **(F)** The bag is removed through the introduction sheath. **(G)** Tissue and fluid remnants after morcellation in the NMC system. **(H)** 1,000-mL methylene blue solution is used to identify eventual system integrity.

The key steps of the surgical procedure involving the NMC system are illustrated in Figure 3 and are also depicted in the Supplementary Video 1. The first stage was placement, and the steps involved are outlined hereafter: (a) the bag was placed in the abdominal cavity through the right lower 20-mm introduction sheath, (b) the myoma was resected into the bag via its large opening, (c) the edges around the opening of the bag were pulled out through the introduction sheath, (d) the hard sheaths on the bag were pulled from the body through the corresponding trocar incisions, (e) the sealing caps were replaced with the trocar bases, (f) the abdominal cavity was de-sufflated, and simultaneously, a pseudo-pneumoperitoneum (14 mmHg) was established by inflating the bag via the umbilical trocar. Thereafter, power morcellation was performed.

The next stage was extraction, and the steps involved are outlined hereafter: (a) after morcellation, the trocar bases were replaced with the sealing caps and sent back into the abdominal cavity, (b) the bag was de-sufflated, and the abdominal cavity was re-insufflated, and (c) the bag was removed through the introduction sheath.

After LM, the abdomen and pelvis were carefully examined for signs of tissue spillage. Surgeons visually examined the integrity of the system. The system was filled with 1,000 mL of methylene blue solution to identify potential disruptions. Precautions were outlined such that in case a system tear or leakage occurred at any time prior to or during the morcellation, the procedure would be halted and a new system would be utilized.

All statistical analyses were performed using SPSS version 31.0 (IBM Corp., USA). Continuous data are summarized as means and standard deviations (SDs). The *t*-test was used for comparing continuous variables and the χ^2^ test or the Fisher's exact test was used for comparing the categorical variables between the unmatched groups. Propensity score matching (PSM) was used to minimize bias; it has been widely used in previous studies as well (16). Based on previous reports and experience, the age; BMI; gravidity and parity; preoperative diabetes and hypertension status; hemoglobin level; ASA score; main surgical indications; history of abdominal/pelvic surgery; and the tumor size, number, and location were considered to be important factors associated with the perioperative outcomes in the two groups. We calculated a propensity score for each patient through logistic regression modeling; thereafter, patients from the study and control groups were matched at a ratio of 1:1, with the caliper width set as 0.02 for the SD. The standardized mean difference was estimated before and after matching to evaluate the balance. Patient demographic data were adjusted to almost the same levels after matching. For proportional outcome comparisons between the two groups after PSM, the paired *t*-test was used for continuous variables and the McNemar test was used for binary variables. Two-sided *p*-values < 0.05 were considered to indicate statistical significance. The analysis was conducted in August 2021.

This study was approved by the institutional review board of the China-Japan Friendship Hospital. All patients were fully informed of the operation; they agreed to undergo it and consented to the further utilization of the data collected before and after the operation. Before the operation, all patients were informed in detail about the operative procedures, potential risks, and benefits of the intervention. The patients had the right to choose whether to undergo treatment using the NMC system, mainly based on their desires. Written informed consent was obtained from all the patients.

## Results

The baseline characteristics of the patients are displayed in Table 1. No significant intergroup differences in most of the baseline characteristics, except for gravidity, average diameter of the largest tumor, and main surgical indications, were observed. After PSM, 386 women were successfully matched such that the previously mentioned baseline differences were no longer present.

**Table 1:** Patient characteristics before and after propensity score matching.

	**Propensity score matching, no. (%)**					
	**Before**			**After**		
**Characteristic**	**Study group** **(*n* = 193)**	**Control group** **(*n* = 1753)**	***P*** **-value**	**Study group** **(*n* = 193)**	**Control group** **(*n* = 193)**	***P*** **-value**
Age, mean (SD), y	39.2 (6.4)	39.2 (7.0)	0.993	39.2 (7.0)	38.7 (6.6)	0.428
BMI			0.134			0.698
<19	17 (8.8)	141 (8.0)		17 (8.8)	21 (10.9)	
19–24	117 (60.6)	946 (54.0)		117 (60.6)	119 (61.7)	
>24	59 (30.6)	666 (38.0)		59 (30.6)	53 (27.5)	
Gravidity, mean (SD)	1.4 (1.4)	1.8 (1.5)	<0.001	1.4 (1.4)	1.6 (1.5)	0.292
Parity, mean (SD)	0.6 (0.6)	0.7 (0.7)	0.034	0.6 (0.6)	0.7 (0.6)	0.654
ASA score			0.060			0.335
1	161 (83.4)	1,555 (88.7)		161 (83.4)	158 (81.9)	
2	32 (16.6)	190 (10.8)		32 (16.6)	35 (18.1)	
≥3	0 (0)	8 (0.5)		0	0	
Diabetes			0.099			1.000
Yes	4 (2.1)	84 (4.5)		4 (2.1)	3 (1.6)	
No	189 (97.9)	1,669 (95.2)		189 (97.9)	190 (98.4)	
Hypertension			1.000			0.724
Yes	3 (1.6)	31 (1.8)		3 (1.6)	5 (2.6)	
No	190 (98.4)	1,722 (98.2)		190 (98.4)	188 (97.4)	
Hemoglobin level			0.630			0.554
Normal	169 (87.6)	1,560 (89.0)		169 (87.6)	164 (85.0)	
Abnormal	24 (12.4)	193 (11.0)		24 (12.4)	29 (15.0)	
Major indications			0.006			0.555
Menorrhagia	675 (38.5)	675 (38.5)		88 (45.6)	100 (51.8)	
Intestinal and urinary tract disorders	174 (9.9)	174 (9.9)		30 (15.5)	21 (10.9)	
Abdominal distention or pain	114 (6.5)	114 (6.5)		13 (6.7)	13 (6.7)	
Fertility requirements	666 (38.0)	666 (38.0)		54 (28.0)	54 (28.0)	
Others	124 (7.1)	124 (7.1)		8 (4.1)	5 (2.6)	
History of abdominal/pelvic surgery			0.376			0.498
No	135 (69.9)	1,169 (66.7)		135 (69.9)	142 (73.6)	
Yes	58 (30.1)	584 (33.3)		58 (30.1)	51 (26.4)	
Myomectomy	8	75		8	7	
Cesarean section	43	384		43	37	
Others	14	178		14	12	
The average diameter of the largest tumor, mean (SD), cm	7 (1.9)	6.7 (2.1)	0.001	7 (1.9)	6.8 (2.0)	0.147
Location of the tumor			0.590			1.000
Uterus	176 (91.2)	1,626 (92.8)		176 (91.2)	176 (91.2)	
Cervical or uterus ligaments	17 (8.8)	123 (7.0)		17 (8.8)	17 (8.8)	
Others	0 (0)	4 (0.2)		0 (0)	0 (0)	
Number of the tumor	2.7 (2.5)	2.5 (2.2)	0.521	2.7 (2.5)	2.8 (2.5)	0.618

*BMI, body mass index; Normal hemoglobin level is ≥ 110 g/L*.

The perioperative outcomes of the two groups are displayed in Table 2. All operations were completed in the study group. In two cases, bag damages were detected before morcellation and the NMC systems were changed. Following PSM, no intraoperative complications were noted in the two groups. Furthermore, no significant differences in the postoperative complications were noted between the two groups (*p* = 1.000; Table 3). There was no significant difference in the mean total operative time between the study and control groups (119.9 ± 46.4 vs. 120.2 ± 44.2 min, *p* = 0.953). The difference in the estimated blood loss per patient was not statistically significant between the study and control groups (73.7 ± 102.7 mL vs. 65.7 ± 76.2 mL, *p* = 0.621). Additionally, there was no significant difference in the postoperative hospitalization duration between the study and control groups (3.8 ± 1.3 vs. 4.0 ± 1.4, *p* = 0.442; Table 2).

**Table 2:** Perioperative outcomes of the two groups.

	**Propensity score matching, no. (%)**					
	**Before**			**After**		
**Characteristic**	**Study group** **(*n* = 193)**	**Control group** **(*n* = 1,753)**	***P*** **-value**	**Study group** **(*n* = 193)**	**Control group** **(*n* = 193)**	***P*** **-value**
Leakage	0	–	–	0	–	–
Intra-op. complications	0	4	–	0	0	–
Post-op. complications	9 (4.7)	99 (5.6)	0.625	9 (4.7)	10 (5.1)	1.000
Total operative time, mean (SD), min	119.9 (46.4)	116.6 (46.7)	0.349	119.9 (46.4)	120.2 (44.2)	0.953
Estimated blood loss, mean (SD), ml	73.7 (102.6)	66.2 (91.8)	0.271	73.7 (102.7)	65.7 (76.2)	0.621
Postoperative hospitalization duration, mean (SD), d	3.8 (1.3)	3.6 (1.3)	0.004	3.8 (1.3)	4.0 (1.4)	0.442

**Table 3:** Peri-operation complications of the two groups.

	**Propensity score matching, no. (%)**					
	**Before**			**After**		
	**Study group (*n* = 193)**	**Control group (*n* = 1,753)**	***P*** **-value**	**Study group (*n* = 193)**	**Control group (*n* = 193)**	***P*** **-value**
Intra-op. complications	*n* = 0	*n* = 4 (0.2) Bladder injury 1 AWV injury 1 EBL > 1,000 mL 2	–	*n* = 0	*n* = 0	–
Post-op. complications	*n* = 9 (4.7) Incisional seroma 1 Incisional infection 1 Urinary tract infection 2 Hematuria 1 Uroschesis 2 Abdominal dressing allergy 2	*n* = 99 (5.6) Blood transfusion 5 Phlebothrombosis 1 Pelvic infection 2 Incisional seroma 12 Incisional infection 15 Subcutaneous emphysema 8 Urinary tract infection 17 Hematuria 11 Uroschesis 15 Abdominal dressing allergy 13	0.625	*n* = 9 (4.7) Incisional seroma 1 Incisional infection 1 Urinary tract infection 2 Hematuria 1 Uroschesis 2 Abdominal dressing allergy 2	*n* = 10 (5.1) Incisional seroma 2 Incisional infection 1 Urinary tract infection 1 Hematuria 1 Uroschesis 2 Abdominal dressing allergy 3	1.000

*EBL, estimated blood loss; AWV, abdominal wall vascular*.

There were no significant differences in the initial pathological types between the two groups (*p* = 0.414; Table 4). Unexpected malignant uterine leiomyosarcoma (ULSM) was diagnosed in one patient (1/193, 0.5%) in the study group. In the control group, five patients (5/1,753, 0.3%) developed malignancy after LM; this included one endometrial stromal sarcoma and four ULSMs. Reoperations for the unexpected sarcomas included laparoscopic hysterectomy and bilateral salpingectomy (LH + BS) or laparoscopic hysterectomy and bilateral salpingo-oophorectomy (LH + BSO) (Table 5).

**Table 4:** Initial pathology types of the two groups.

	**Pathology types**	**Study group** **(*n* = 193) No. (%)**	**Control group (*n* = 1,753) No. (%)**	***P*** **-vaule**
Initial pathological types	UL	143 (74.1)	1,259 (71.8)	0.414
	Special types of UL^a^	49 (25.4)	489 (27.9)	
	Uterine sarcoma	1 (0.5)	5 (0.3)	

*UL, uterine leiomyoma*.

a *Special types uterine leiomyoma including atypical leiomyomas, cell-rich leiomyomas, and leiomyomas with uncertain malignant potential, et al*.

**Table 5:** Patients with unexpected uterine sarcoma during laparoscopic surgeries for uterine leiomyoma.

**Groups**	**Patients**	**Age**	**Pathology**	**FIGO** **stage**	**Supplementary treatment**	**Peritoneal dissemination** **(Time to the first operation)**	**Follow-up time (months)**	**Survival state**
Study group	1	26	ULMS	Ia	LH + BS	No	24	Survival
Control group	1	32	ULMS	Ia	LH + BSO	No	62	Survival
	2	36	ESS	Ib	LH + BSO	Yes (2 months)	25	Die
	3	41	ULMS	Ia	LH + BSO	No	72	Survival
	4	41	ULMS	Ia	LH + BSO	Yes (5 months)	73	Survival
	5	43	ULMS	Ia	LH + BSO	Yes (6 months)	71	Survival

*FIGO, The International Federation of Gynecology and Obstetrics; ULMS, uterine leiomyosarcoma; ESS, endometrial stromal sarcoma; LH + BS, laparoscopic hysterectomy; bilateral salpingectomy; LH + BSO, laparoscopic hysterectomy, bilateral salpingo-oophorectomy*.

The median follow-up times in the study and control groups were 21 months (range: 12–36 months) and 54 months (range: 12–92 months), respectively. No peritoneal tissue spread was found in the study group. However, 2–6 months after LM, three patients (3/5, 0.6%) in the control group experienced pathologically confirmed sarcoma peritoneal spread. Furthermore, six patients (6/1,753, 0.3%) developed a benign peritoneal tissue spread [the lesions included parasitic leiomyoma and disseminated peritoneal leiomyomatosis (DPL)] due to the recurrence of myomas, abdominal pain, or other reasons. The interval to subsequent surgery ranged from 31 to 73 months (Table 6).

**Table 6:** Power morcellation-related reoperation of benign pathologies in the control group.

**Control group**	**Patients**	**Age** **(years)**	**Initial pathology**	**Interval to subsequent surgery (months)**	**Site**
Parasitic leiomyoma	1	20	Leiomyoma	31	Pelvic leiomyoma
	2	35	Leiomyoma	33	Pelvic leiomyoma
	3	41	Leiomyoma	66	Pelvic leiomyoma
	4	37	Cell-rich leiomyoma	54	Trocar site of the previous LM
DPL	5	33	STUMP	36	Peritoneum, Omentum,
	6	36	Leiomyoma	73	Peritoneum, Omentum, Mesocolon

*LM, laparoscopic myomectomy; DPL, disseminated peritoneal leiomyomatosis*.

## Discussion

The use of containment systems in power morcellation is accepted worldwide to thwart the spread of occult malignant tumors (5, 6). However, owing to the poor compatibility between the extraction bag and the surgical instrument, the bag has a high leakage rate and is not easy to operate. This limits the development of minimally invasive surgery (11–14). Therefore, in this study, we reported an NMC system with the aim of realizing the integration of the containment bag and laparoscopic trocars to form a completely sealed containment barrier that prevents the spillage of liquids and tissue from the time the uterine tissue is morcellated and encapsulated in the system.

Several protected techniques by using endobag systems for UL power morcellation have been described in the literatures (17–27). Although these technologies can play a protective role, there are still three major shortcomings. First, the soft auxiliary sleeve is easily twisted and punctured when the surgical instruments enter the bag through a small incision. Second, the end of the auxiliary sleeve is sealed by knotting or suture ligation during removal through the abdominal wall incision; this is inadequate for preventing the sealing area from being contaminated by the tissue. Furthermore, when the bag is removed through the incision, an excessive pressure is generated inside the bag; this puts the sealed area at the highest risk of leakage. Moreover, it is inconvenient to operate by a single port or without the help of an assistant; this does not conform to the traditional laparoscopic operating habits and may increase the difficulty and risk of operation. In addition, using endobag inserted through the posterior colpotomy to remove the specimen is also an effective and feasible alternative technique, and has better a cosmetic effect (28–30). Nevertheless, transvaginal procedures cannot be performed in patients without a sexual history. Furthermore, it is extremely difficult to apply in patients with a narrow vaginal capacity, an obliterated cul-de-sac, or in whom larger myomas require removal (30).

To solve these problems, the system makes use of a hard sheath and sealing cap; these are connected through a reliable and stable threaded interface, thus providing good protection against the spread of tumors. The auxiliary sleeve of the system is composed of a hard material that effectively prevents the twisting or puncturing of the bag. In addition, a sealing cap is used to seal the hard sheath, which can maintain the integrity of the system during the extraction process. The uterine tissue in the bag is completely covered by the sealing cap and does not come in contact with the abdominal wall incision; compared with suture ligation or knotting, the threaded connection can provide tighter and more pressure-resistant protection to the end of the auxiliary sleeve.

In our study, bag damage occurred in two cases in the study group; however, in both cases, the damage was discovered before morcellation, and the NMC systems were replaced. The postoperative methylene blue solution test confirmed that there was no leakage. These two cases of damage occurred in the first 30 cases, and the damaged parts were near the small hard sheath. We considered that this may be due to a torsion between the rigid sheath and the bag body. We also considered that the laparoscopic instrument may have been introduced when not sufficiently adjusted, which may have caused the bag to puncture. In subsequent operations, full attention was paid to the procedure, and all the operations were performed under direct laparoscopic view; therefore, no damage occurred.

On analyzing the follow-up data in the control group, we identified three patients with uterine sarcomas who suffered intraperitoneal dissemination soon after LM (2–6 months); the spread rate was 0.6% (3/5). This result is consistent with the results of previous studies (31, 32). Unprotected morcellation is detrimental to the patient. In case of a malignant sarcoma, it will cause disease upstaging and negatively affect the prognosis. Only one patient in the study group had a ULSM; it has been followed up for 24 months with no evidence of dissemination. In addition to malignancy, there is a risk of benign tissue spread after unprotected morcellation (5, 18, 33, 34). Four cases were observed in the control group and none in the study group. Although there were certain discrepancies in the sample scale and follow-up time between the two groups, because the NMC system has an airtight protective effect and there was no damage or leakage during the operation, we believe that the probability of disseminated implantation is extremely low. Of course, a longer follow-up time is needed for further verification, especially of dissemination of benign tissues.

Finally, to ease manipulation, we used the multi-port rigid sheaths as auxiliary ports, because they can match with the multi-port laparoscope to facilitate the operation with the help of an assistant.

Some studies have reported that the application of the endobag system could be time-consuming due to bag manipulation (27). Based on our findings, the total operative time did not differ significantly between the study and control groups. The reason may be that although the placement and extraction of the system spend more time, the time in searching for tissue fragments and performing repeat irrigation after morcellation can be saved, and the multi-port rigid sheath makes the operation easier.

There were some limitations in our study. The main limitations were the bias associated with patient selection and the retrospective nature of the study. Therefore, to provide stronger evidence, further prospective, multicenter, large-sample clinical studies (NCT 04392674) will be performed. Besides, the control group did not receive any protection during morcellation; thus, there was a risk of tissue dissemination. Although the U.S. FDA proposed a prohibition of power morcellation in 2014; in 2020, it updated the guidelines again to point out the need for morcellation containment system (9, 10). In fact, it was not until 2020 that China proposed the consensus on the implementation of laparoscopic power morcellation for UL (35). During this period, there were some disputes (36). Furthermore, in China, there are still great differences in the economic levels. Many protective devices have not been included in the reimbursement of basic medical insurance; this exerts additional economic burden on the patients. Therefore, some patients still take a chance to choose uncontained morcellation despite knowing the relevant risks; however, the number of patients who make such a choice has reduced significantly. In recent years, Chinese gynecologist have been making continuous efforts to ensure the safety of LM. Furthermore, apart from us, other research groups in China, are also investigating some self-made protective bags during morcellation. However, in general, we are still in the exploratory stage about the containment systems. We believe that the use of containment systems will prevent the risk of tumoral spread caused by power morcellation.

In conclusion, the NMC system is compatible with traditional laparoscopy and can easily comminute fully enclosed fibroids in the abdominal cavity. It is safe, effective, and feasible for the dissemination of tumors under laparoscopy.

## Data Availability Statement

The raw data supporting the conclusions of this article will be made available by the authors, without undue reservation.

## Ethics Statement

The studies involving human participants were reviewed and approved by the Institutional Review Board of the China-Japan Friendship Hospital. The patients/participants provided their written informed consent to participate in this study.

## Author Contributions

WW, WF, YD, and MX: statistical analysis. BL, JL, DF, HL, HY, and FZ: obtained funding. JL, BL, FZ, HY, QC, CR, and YY: administrative, technical, or material support. WW, JL, and BL: supervision. All authors: critical revision of the manuscript for important intellectual content.

## Funding

This work was supported by the China-Japan Friendship Hospital Funds (Nos. 2017-RC-4 and 2019-2-MS-2).

## Conflict of Interest

The authors declare that the research was conducted in the absence of any commercial or financial relationships that could be construed as a potential conflict of interest.

## Publisher's Note

All claims expressed in this article are solely those of the authors and do not necessarily represent those of their affiliated organizations, or those of the publisher, the editors and the reviewers. Any product that may be evaluated in this article, or claim that may be made by its manufacturer, is not guaranteed or endorsed by the publisher.
